# Computational data of phytoconstituents from *Hibiscus rosa-sinensis* on various anti-obesity targets

**DOI:** 10.1016/j.dib.2019.103994

**Published:** 2019-05-16

**Authors:** Sejal P. Gandhi, Kiran B. Lokhande, Venkateswara K. Swamy, Rabindra K. Nanda, Sohan S. Chitlange

**Affiliations:** a Department of Pharmaceutical Chemistry, Dr. D. Y. Patil Institute of Pharmaceutical Sciences and Research, Pimpri, Pune, India; b Bioinformatics Research Laboratory, Dr. D. Y. Patil Biotechnology & Bioinformatics Institute, Dr. D. Y. Patil Vidyapeeth, Tathawade, Pune, India

## Abstract

Molecular docking analysis of twenty two phytoconstituents from *Hibiscus rosa-sinensis*, against seven targets of obesity like pancreatic lipase, fat and obesity protein (FTO protein), cannabinoid receptor, hormones as ghrelin, leptin and protein as SCH1 and MCH1 is detailed in this data article. Chemical structures of phytoconstituents were downloaded from PubChem and protein structures were retrieved from RCSB protein databank. Docking was performed using FlexX software Lead IT version 2.3.2; Bio Solved IT. Visualization and analysis was done by Schrodinger maestro software. The docking score and interactions with important amino acids were analyzed and compared with marketed drug, orlistat. The findings suggest exploitation of best ligands experimentally to develop novel anti-obesity agent.

**Table:** Specifications table

Subject area	Chemistry
More specific subject area	Computational chemistry
Type of data	Table, figure
How data was acquired	Ligand based molecular docking using FlexX and Maestro software
Data format	Raw and analyzed
Experimental factors	Phytoconstituents structures downloaded from PubChem were subjected to Avogadro software for energy minimization.
Experimental features	Minimized ligands structures were docked with seven selected protein structure using FlexX software.
Data source location	Department of bioinformatics, Dr. D. Y. Patil Biotechnology & Bioinformatics Institute Tathawade, Pune
Data accessibility	Data is only with this article
Related research article	K. H. Min, J. Yoo, H. Park, Computer-Aided Identification of Ligands for GPCR Anti-Obesity Targets, Curr Top Med Chem. 9 (2009) 539–553 [1].

**Table:** 

**Value of the data** • Obesity declared as a disease by WHO and is the main cause of other many metabolic disorders which lead to mortality. • Literature explains multiple mechanisms involved in energy uptake and energy consumption, the control of which can help in maintaining energy balance and thus keeping obesity at large. • This article provides all dataset of protein structures to explore potential targets for obesity. • In-silico exploration of targets is the first step in drug design to understand the underlying mechanism of action of the identified drug molecule. • Many herbal medicines and food supplements are found to be beneficial in reducing body weight, although mode of action and identification of marker phytoconstituents is still not explored. • Docking of phytoconstituents to seven identified targets for obesity can pave a way towards identification of novel anti-obesity drug.

## 1 Data

**Fig. 1:**
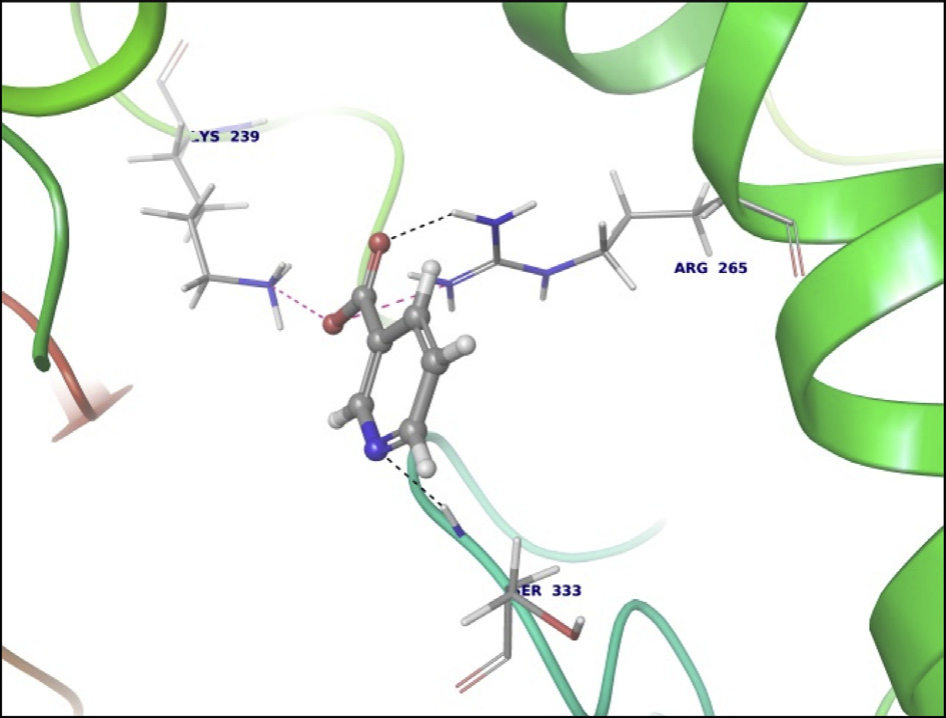
1LPB interaction with Niacin.

**Fig. 2:**
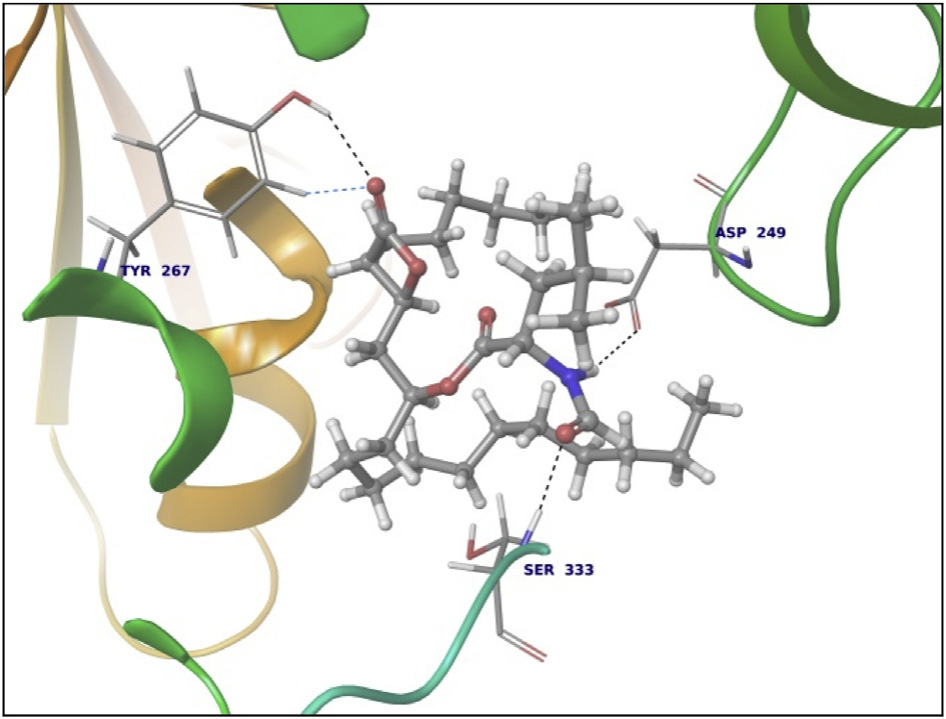
1LPB interaction with Orlistat.

**Fig. 3:**
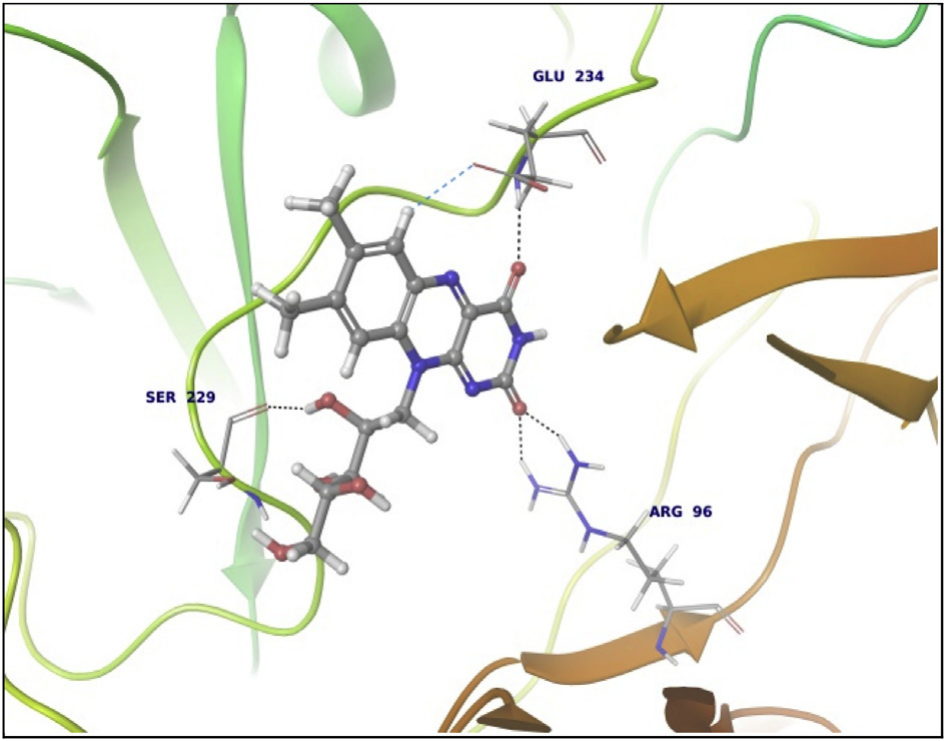
3LFM interaction with Riboflavin.

**Fig. 4:**
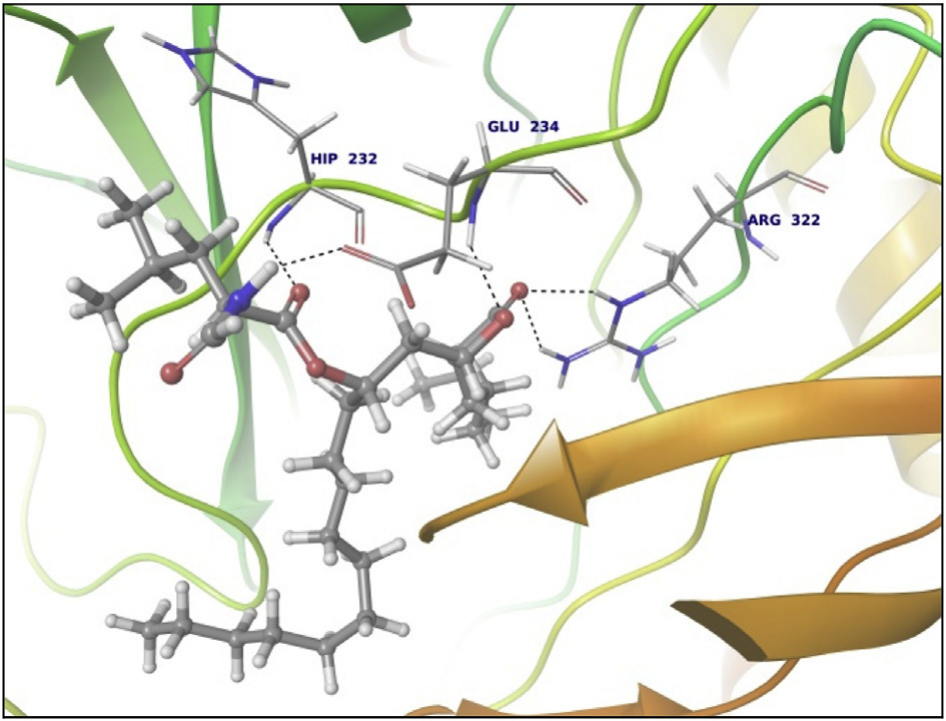
3LFM interaction with Orlistat.

**Fig. 5:**
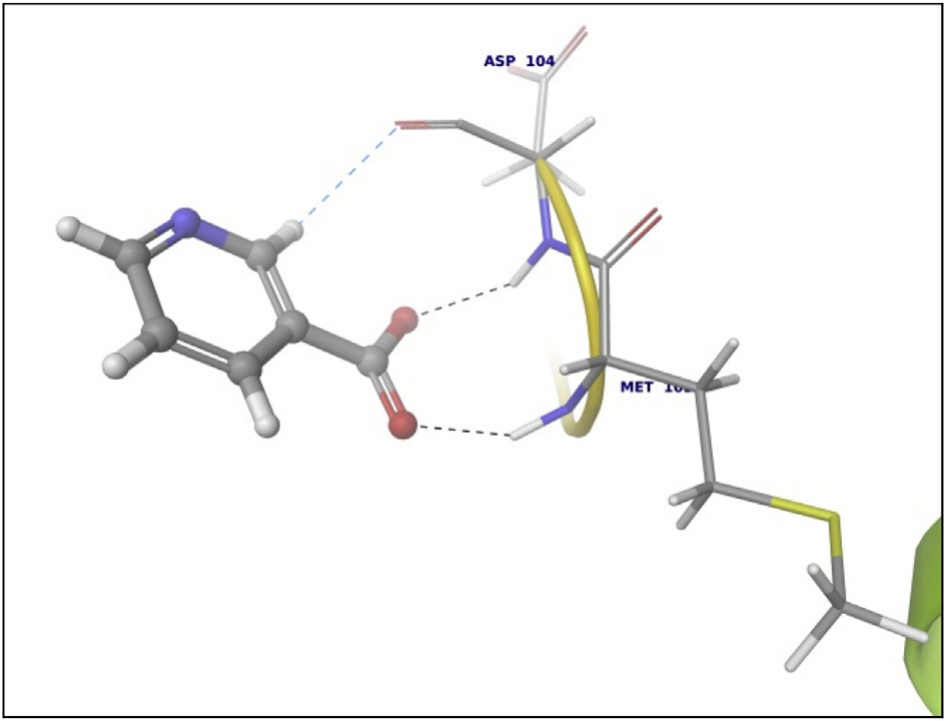
3TGZ interaction with Niacin.

**Fig. 6:**
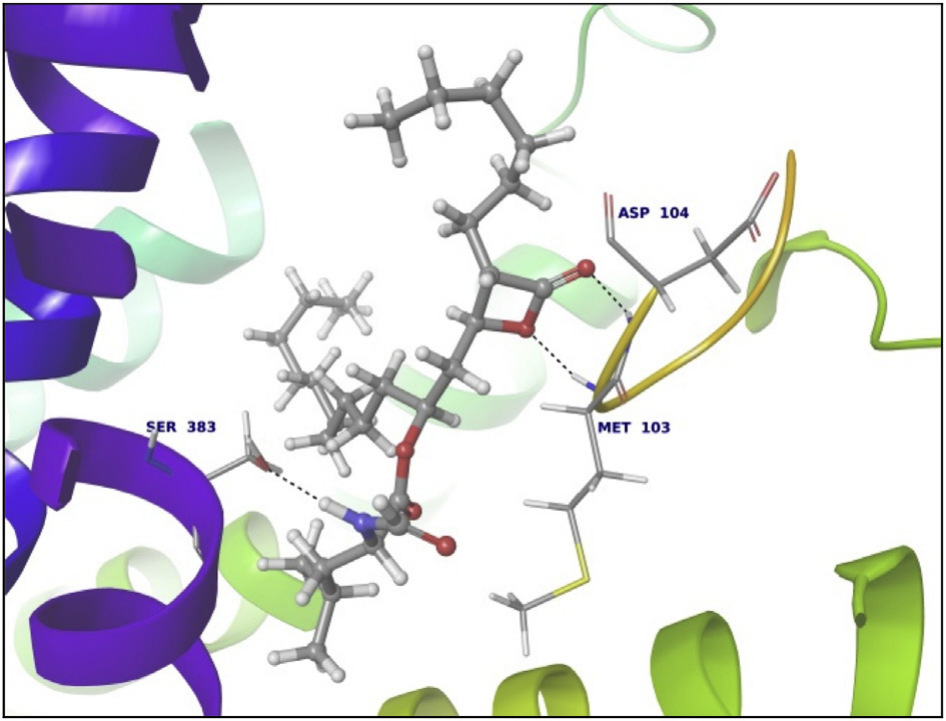
3TGZ interaction with Orlistat.

**Fig. 7:**
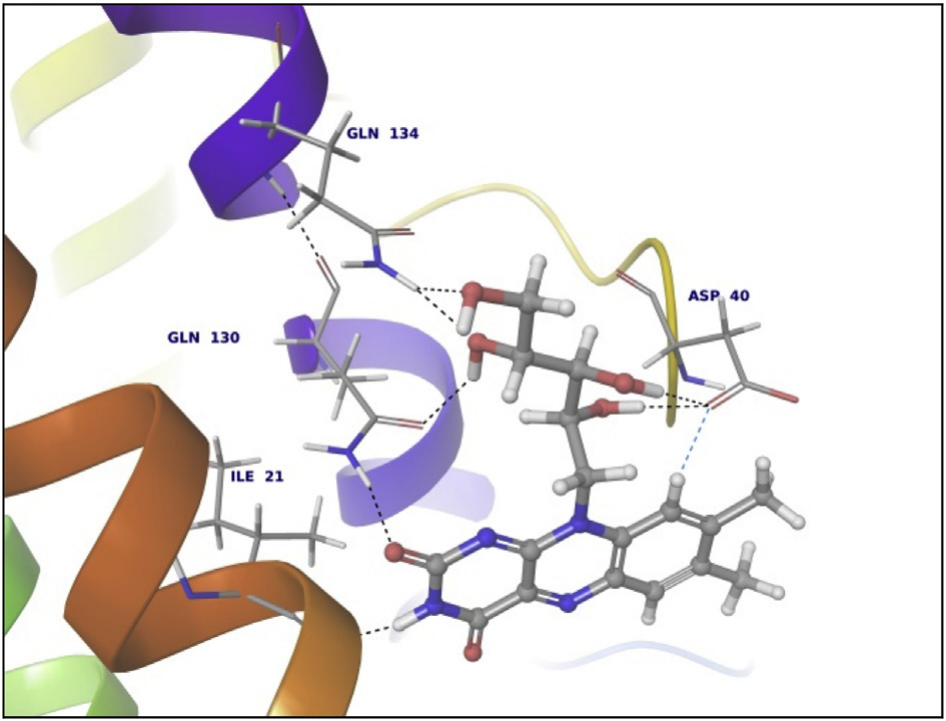
1AX8 interaction with Riboflavin.

**Fig. 8:**
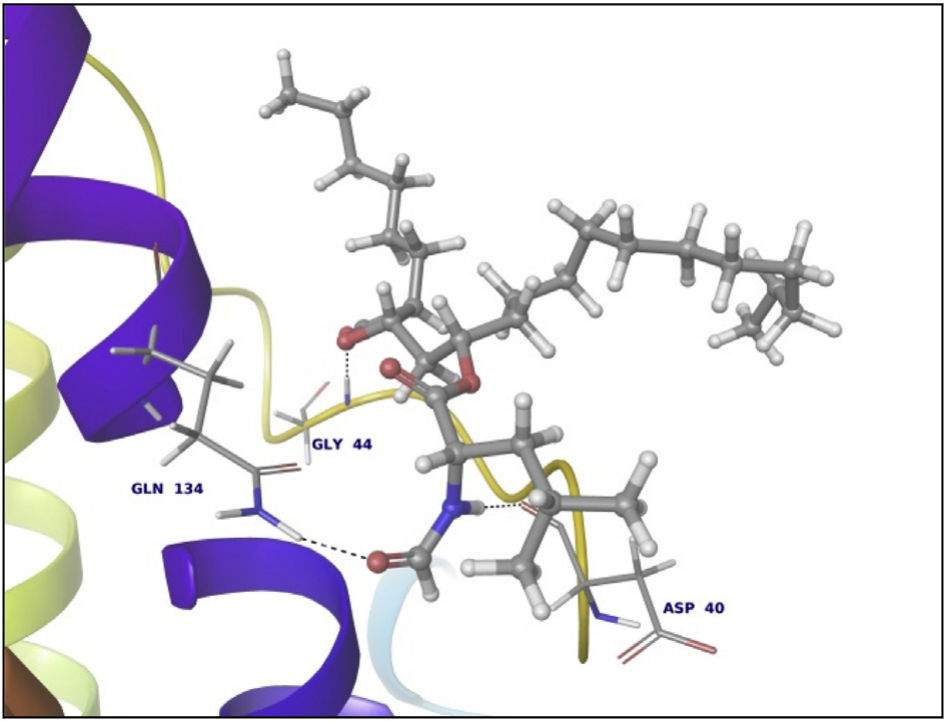
1AX8 interaction with Orlistat.

**Fig. 9:**
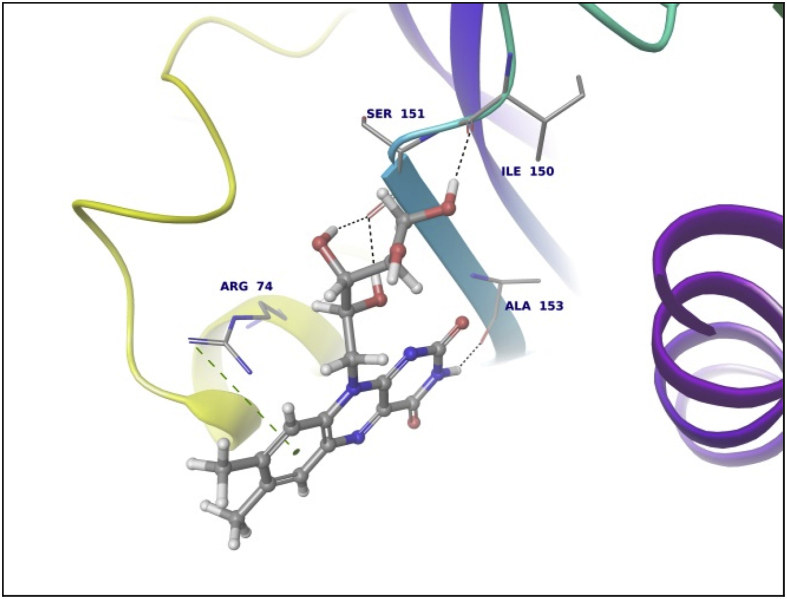
4XWX interaction with Riboflavin.

**Fig. 10:**
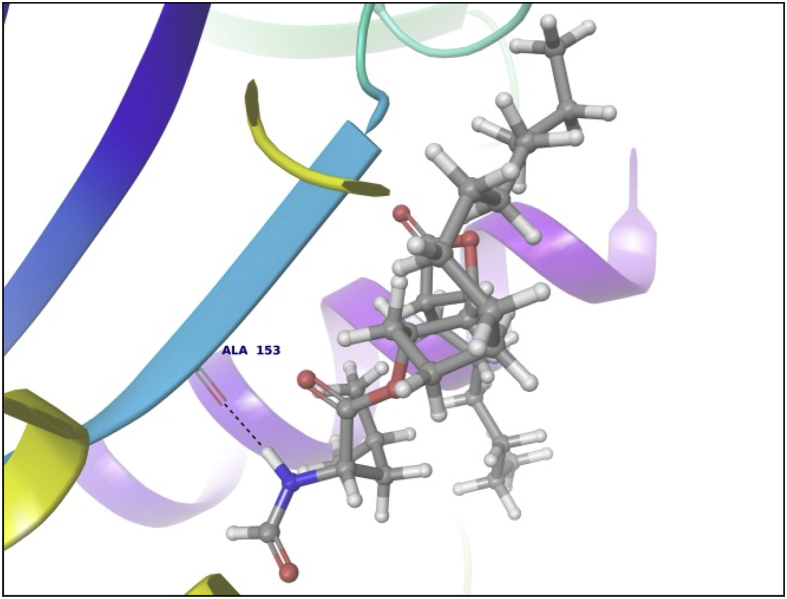
4XWX interaction with Orlistat.

**Fig. 11:**
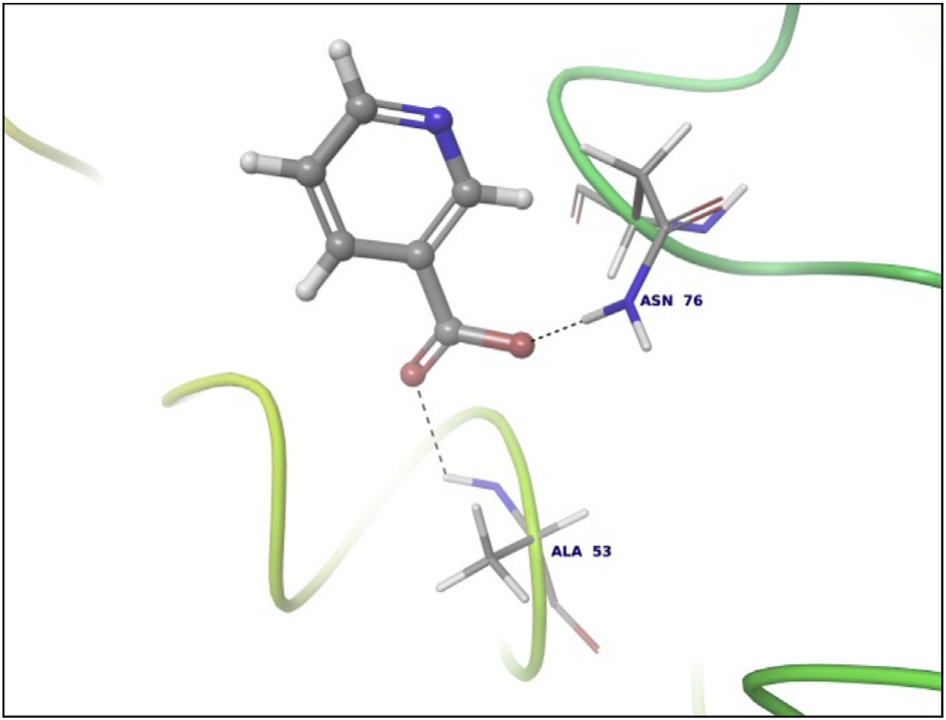
Ghrelin interaction with Niacin.

**Fig. 12:**
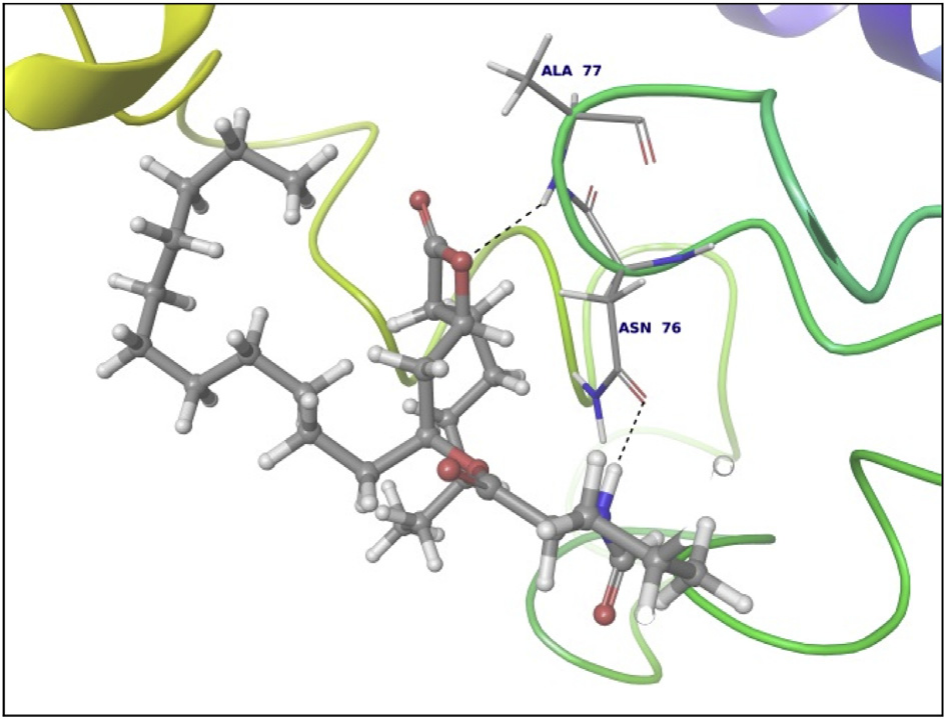
Ghrelin interaction with Orlistat.

**Fig. 13:**
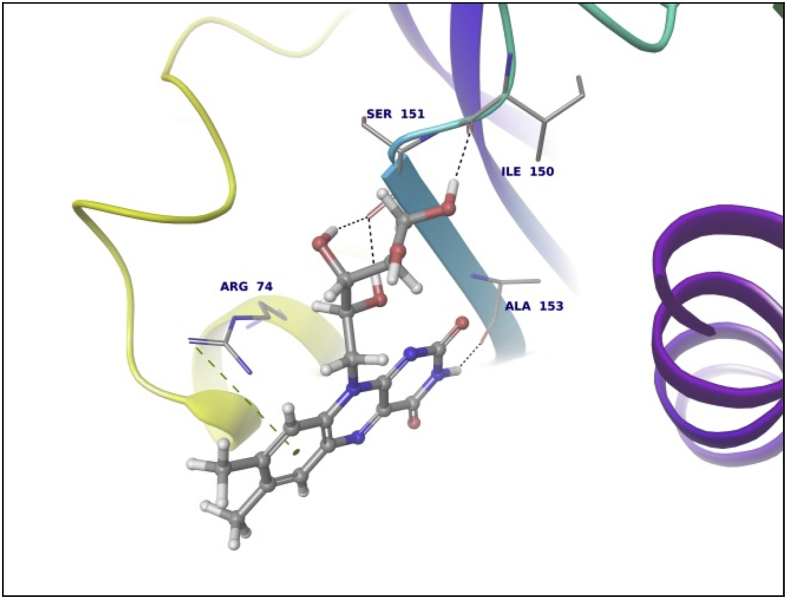
MCH1 interaction with Riboflavin.

**Fig. 14:**
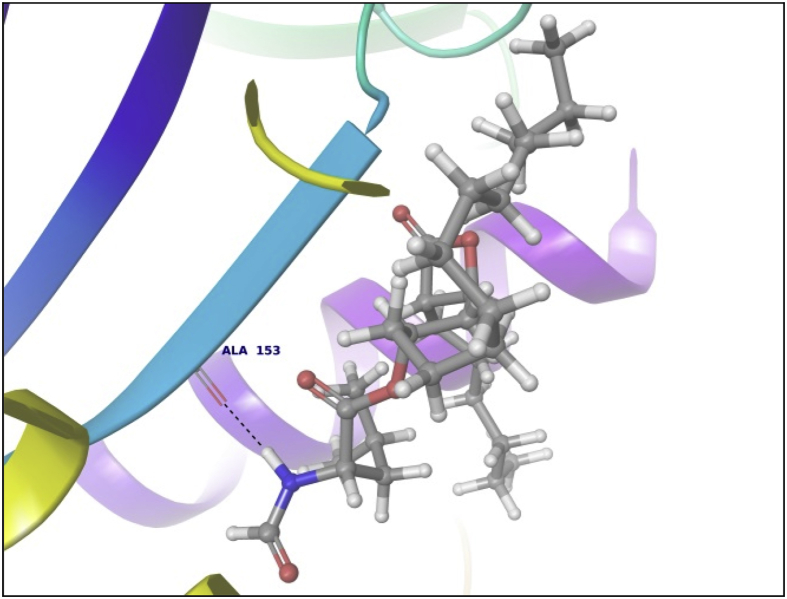
MCH1 interaction with Orlistat.

This dataset contains docking analysis of phytoconstituents of *Hibiscus rosa-sinensis* on different targets of obesity. Different secondary metabolites present in *Hibiscus rosa-sinensis* were selected. Chemical structures of selected phytoconstituents were taken from database and were subjected to energy minimization. Seven receptor structures were selected as potential targets of obesity [1], [2], [3], [4], [5], [6], [7], [8]. Protein structures available in database were downloaded from RCSB protein databank. Table 1 gives details of the selected receptors. Two receptors model were prepared using I-TASSER server online. Table 2 summarizes FASTA sequence of Ghrelin and MCH1 receptor subjected to model preparation. Phytoconstituents were docked on the above targets to understand binding interactions. Table 3, Table 4, Table 5, Table 6, Table 7, Table 8, Table 9 summarizes the dock score, bond distance and interacting amino acid residue of all phytoconstituents on seven different targets. Fig. 1-14 gives docked images of phytoconstituents with lowest dock score and standard drug orlistat with seven receptor proteins.

**Table 1:** Table summarizing details of targets selected.

Target	PDB ID	Description	Resolution	R value free	R value work
Pancreatic Lipase	1LPB	The 2.46 Å resolution structure of the pancreatic lipase colipase complex inhibited by a c11 alkyl phosphonate	2.46 Å	0.285	0.183
Fat And Obesity Protein	3LFM	Crystal structure of the fat mass and obesity associated (FTO) protein reveals basis for its substrate specificity	2.5 Å	0.285	0.239
Cannabinoid Receptor	5TGZ	Crystal structure analysis of w35f/h207w mutant of human clic1	2.3 Å	0.306	0.240
Leptin	1AX8	Human obesity protein, leptin	2.4 Å	0.283	0.185
SCH1 Protein	4XWX	Crystal structure of the PTB domain of SHC	1.87 Å	0.191	0.168

**Table 2:** Uniprot ID and FASTA sequence of ghrelin and MCH1 receptor.

Target	UniProt ID	FASTA sequence
Ghrelin receptor	Q9UBU3	MPSPGTVCSLLLLGMLWLDLAMAGSSFLSPEHQRVQQRKESKKPPAKLQPRALAGWLRPEDGGQAEGAEDELEVRFNAPFDVGIKLSGVQYQQHSQALGKFLQDILWEEAKEAPADK
MCH 1 receptor	Q99705	MSVGAMKKGVGRAVGLGGGSGCQATEEDPLPNCGACAPGQGGRRWRLPQPAWVEGSSARLWEQATGTGWMDLEASLLPTGPNASNTSDGPDNLTSAGSPPRTGSISYINIIMPSVFGTICLLGIIGNSTVIFAVVKKSKLHWCNNVPDIFIINLSVVDLLFLLGMPFMIHQLMGNGVWHFGETMCTLITAMDANSQFTSTYILTAMAIDRYLATVHPISSTKFRKPSVATLVICLLWALSFISITPVWLYARLIPFPGGAVGCGIRLPNPDTDLYWFTLYQFFLAFALPFVVITAAYVRILQRMTSSVAPASQRSIRLRTKRVTRTAIAICLVFFVCWAPYYVLQLTQLSISRPTLTFVYLYNAAISLGYANSCLNPFVYIVLCETFRKRLVLSVKPAAQGQLRAVSNAQTADEERTESKGT

**Table 3:** Summary of docking analysis with pancreatic lipase (PDB ID 1LPB).

Sr. No	Posename	Score	Interacting Residues	Bond Type	Bond Distance
1	Niacin	−27.2868	SER 333	HB	2.01
			ARG 265	Pi-Pi Stacking	5.21
				HB	1.79
				Salt bridge	3.08
			LYS 239	Salt bridge	2.73
2	Quercetin 3, 7 diglucoside	−21.223	LYS 239	HB	1.93
			ASP 247	HB	1.93
			ASP 257	HB	1.71
			TRY 267	HB	1.98
			THR 271	HB	2.70
			LYS 268	HB	1.48
				Pi cation	5.10
			ASP 249	HB	2.12
3	Ascorbic acid	−20.6315	SER 333	HB	2.32
			ASP 247	HB	2.06
			ARG 265	HB	2.18
			ASP 257	HB	2.17
			ASP 249	HB	1.79
4	Quercetin 3, 3′ diglucoside	−20.3198	SER 333	2HB	2.12,2.31
			ASP 247	HB	2.34
			ASP 331	HB	1.84
			ARG 265	HB	2.20
			ASP 257	HB	2.66
			TYR 267	HB	2.2.3
			ASN 88	HB	2.52
5	Quercetin 3,4′ diglucoside	−18.4448	ASP 331	2HB	2.18, 2,07
			ARG 265	HB	1.90
			SER 333	HB	2.29
			PHE 335	Pi Pi stacking	5.46
			ASN 88	HB	2.61
6	8 nonynoic acid	−17.7764	LYS 239	HB	2.04
				Salt Bridge	3.91
			ARG 265	HB	1.93
7	9 Decynoic acid	−17.4676	ARG 265	2HB	2.11, 1.85
			LYS 239	HB	1.94
				Salt bridge	4.67
8	Cyanidine 3, 5 diglucoside	−15.6327	ARG 265	PI Pi stacking	4.92
			ASP 247	HB	1.91
			ASP 257	HB	1.89
			ASP 249	HB	2.11
				Salt Bridge	4.60
			GLU 253	HB	2,29
			SER 333	HB	2.39
			LYS 268	HB	2.29
			ASP 272	HB	2.08
9	Riboflavin	−15.3182	ASP 249	HB	1.36
			SER 333	HB	1.60
			GLU 253	HB	2.16
			LYS 268	HB	1.71
10	Thiamine	−14.7694	ASP 249	HB	1.97
				Salt Bridge	4.85
			ARG 265	Pi Pi stacking	4.94
			LYS 268	Pi cation	2.86
			ASP 247	HB	2.07
11	Beta rosasterol	−9.4736	LYS 239	HB	2.16
			ARG 265	HB	2.16
12	Cyanidin 3-sophoroside-5-glucoside	−8.2017	ASP 249	3HB	1.46, 2.02, 1.91
			ASP 272	HB	2.02
			GLU 253	2HB	1.82, 1.81
13	Methyl non-8-ynoate	−7.2264	LYS 239	HB	2.05
			ARG 265	HB	1.94
14	Methyl Dec-9-ynoate	−5.9149	LYS 239	HB	2.05
			ARG 265	HB	1.94
15	Methyl (E)-11-methoxy-9-oxononadec-10-enoate	−4.9341	SER 333	HB	2.14
			ARG 265	HB	2.15
			TRY 267	HB	2.14
			LYS 268	HB	2.12
16	Methyl malvalate	−3.6439	LYS 239	HB	2.05
			ARG 265	HB	1.94
17	Methyl 8-oxooctadec-9-ynoate	−2.8512	SER 333	HB	2.10
			LYS 239	HB	1.89
			ARG 265	HB	2.02
18	Methyl Sterculate	−1.1816	ARG 265	HB	1.98
			LYS 239	HB	2.06
19	Campesterol	1.5909	GLU 253	HB	2.28
20	Stigmasterol	2.651	ASP 249	HB	1.90
			GLU 253	HB	2.12
21	Beta sitosterol	3.2084	No interaction		
**22**	**Orlistat**	**0.1075**	**ASP 249**	**HB**	**1.68**
			**SER 333**	**HB**	**1.94**
			**TYR 267**	**HB**	**2.23**
				**Ar HB**	**1.91**

Orlistat, as only standard drug used in market is used as standard reference for docking studies. Hence the docking result of orlistat in all tables is bold for ease of comparison.

**Table 4:** Summary of docking analysis with fat and obesity protein (PDB ID 3LFM).

Sr. No.	Ligand	Score	Interacting Residues	Bond Type	Bond Distance
1	Riboflavin	−27.3248	ARG 96	HB	1.62
			SER 229	HB	2.07
			GLU 234	HB	2.01
				Ar HB	2.35
2	Niacin	−21.5279	ARG 322	HB	1.93
			GLU 234	HB	1.84
			ARG 96	Pi-Pi Stacking	4.26
3	Thiamine	−19.313	TRY 108	Pi-Pi Stacking	4.78
			HIP 231	Pi-Pi Stacking	3.68
				Pi-Pi Stacking	5.45
				Pi Cation	4.42
				Pi Cation	3.78
			SER 229	HB	1.38
			TYR 106	HB	2.00
4	Ascorbic acid	−16.8546	ASP 233	HB	1.99
			ARG 322	HB	2.45
			ARG 96	HB	1.99
			GLU 234	HB	1.90
5	Cyanidine 3, 5 diglucoside	−14.6454	ARG 322	Pi Cation	5.23
				HB	1.52
			TRY 106	HB	2.08
				HB	1.81
			HIP 232	HB	1.82
			GLU 234	HB	2.25
			HIP 231	Pi-Pi Stacking	4.81
				Pi-Pi Stacking	5.43
			VAL 94	HB	1.53
6	Quercetin 3,4′ diglucoside	−12.747	VAL 94	HB	2.35
			GLU 234	HB	2.00
			HIP 232	HB	1.57
				HB	1.91
			GLN 306	HB	2.18
			HIP 231	Pi Cation	6.38
7	8 nonynoic acid	−12.149	ASN 205	HB	1.96
			ARG 322	HB	2.05
				Salt bridge	3.78
			ARG 96	HB	1.78
8	9 Decynoic acid	−11.8069	ARG 322	HB	1.97
			GLU 234	HB	1.97
9	Quercetin 3,3′ diglucoside	−11.2637	TYR 108	Pi-Pi Stacking	4.55
			ARG 96	HB	2.66
			VAL 94	HB	1.79
			ALA 227	HB	2.24
			GLU 234	HB	1.62
10	Quercetin 3,7 diglucoside	−7.7494	GLU 234	HB	2.04
				HB	2.16
			TYR 108	Pi-Pi Stacking	4.94
			TYR 106	HB	2.29
			ARG 322	HB	1.71
			HIP 231	Pi-Pi Stacking	3.98
			HIP 232	HB	2.06
11	Methyl 8-oxooctadec-9-ynoate	−6.5642	HIP 232	HB	1.93
			ARG 96	HB	1.52
12	Methyl Dec-9-ynoate	−4.8041	ARG 96	HB	2.15
13	Methyl non-8-ynoate	−4.4543	ARG 96	HB	2.15
14	(9) Methyl (E)-11-methoxy-9-oxononadec-10-enoate	−2.4721	ARG 96	HB	2.15
15	Beta rosasterol	−1.029	VAL 94	HB	1.78
16	Methyl Sterculate	0.5157	ARG 96	HB	1.84
17	Methyl malvalate	0.7329	ARG 96	HB	1.88
18	Beta sitosterol	1.2521	ALA 227	HB	2.2
19	Campesterol	1.447	ALA 227	HB	2.21
20	**Orlistat**	**−7.2466**	**ARG 322**	**HB**	**2.20**
			**GLU 234**	**HB**	**2.04**
				**HB**	**1.66**
			**HIP 232**	**HB**	**2.17**

Orlistat, as only standard drug used in market is used as standard reference for docking studies. Hence the docking result of orlistat in all tables is bold for ease of comparison.

**Table 5:** Summary of docking analysis with cannabinoid receptor (PDB ID 3TGZ).

Sr. No.	Ligand	Score	Interacting Residues	Bond Type	Bond Distance
1	Niacin	−14.7132	MET 103	HB	1.84
			ASP 104	HB	2.07
2	Thiamine	−13.5476	PHE 102	Pi-Pi Stacking	4.92
			SER 383	HB	1.81
			SER 123	HB	1.69
3	Ascorbic acid	−11.9942	ASP 163	HB	2.30
			TRP 356	HB	1.70
			CYS 386	HB	1.86
			SER 199	HB	2.44
			ALA 162	HB	2.12
4	Riboflavin	−9.4202	PHE 170	Pi-Pi Stacking	5.43
			MET 103	HB	1.96
			SER 383	HB	2.06
5	8 nonynoic acid	−4.3902	ASP 104	HB	1.84
6	9 Decynoic acid	−3.8828	ASP 104	HB	1.95
			MET 103	HB	1.83
7	Methyl 8-oxooctadec-9-ynoate	−3.0906	ASN 389	HB	2.66
			TRP 356	HB	1.86
8	Methyl non-8-ynoate	−2.5398	TRP 356	HB	1.95
9	Methyl Dec-9-ynoate	−2.4934	TRP 356	HB	1.95
10	(9) Methyl (E)-11-methoxy-9-oxononadec-10-enoate	−2.1673	TRP 356	HB	1.95
11	Quercetin 3,3′ diglucoside	−1.505	SER 383	HB	2.61
			TRP 356	HB	2.51
			SER 390	HB	1.50
12	Methyl malvalate	−0.5677	No Interaction		
13	Methyl Sterculate	−0.2554	ASN 389	HB	2.50
			TRP 356	HB	1.85
14	Quercetin 3,4′ diglucoside	−0.201	PHE 174	Pi-Pi Stacking	5.44
			ASP 104	HB	2.14
15	Campesterol	3.5794	No Interaction		
16	Beta rosasterol	6.6198			
17	Beta sitosterol	6.6198			
18	**Orlistat**	**−1.7877**	**MET 103**	**HB**	**1.82**
			**ASP 104**	**HB**	**2.09**
			**SER 383**	**HB**	**1.65**

Orlistat, as only standard drug used in market is used as standard reference for docking studies. Hence the docking result of orlistat in all tables is bold for ease of comparison.

**Table 6:** Summary of docking analysis with leptin (PDB ID 1AX8).

Sr. No.	Ligand	Dock Score	Interacting residues	Bond Type	Bond distance
1	Riboflavin	−18.4869	GLN 134	HB	2.22
				HB	1.91
			GLN 130	HB	2.12
				HB	1.72
			ASP 40	HB	2.08
				HB	1.58
				Ar HB	2.21
			ILE 21	HB	1.80
2	Cyanidine 3, 5 diglucoside	−13.4683	ASP 40	HB	1.49
				HB	2.40
				HB	1.75
				HB	1.68
			GLN 130	HB	1.87
				HB	1.83
			GLN 134	HB	2.41
			ILE 21	HB	1.86
				HB	1.53
3	Thiamine	−11.3807	GLN 134	HB	2.21
			ASP 40	HB	2.15
			ILE 42	HB	1.84
4	Ascorbic acid	−11.1364	GLY 44	HB	1.94
			GLN 134	HB	1.98
				HB	2.20
5	Quercetin 3,4′ diglucoside	−10.9657	GLY 44	HB	2.57
				HB	2.27
			ASP 135	HB	2.15
			GLN 130	HB	1.90
			ASP 40	HB	2.05
			LEU 39	HB	1.84
				HB	1.92
6	Quercetin 3,3′ diglucoside	−10.3108	ASP 40	HB	2.29
			SER 127	HB	1.60
7	Quercetin 3,7 diglucoside	−10.2723	PHE 41	Pi-Pi Stacking	5.04
			GLN 130	HB	1.97
			ASP 40	HB	2.07
			GLY 131	HB	1.56
			GLY 44	HB	1.64
			ASP 135	HB	1.56
8	Niacin	−9.3776	ASP 40	HB	1.84
9	Beta rosasterol	−6.3064	GLY 44	HB	1.82
10	Cyanidin 3-sophoroside-5-glucoside	−5.2426	GLN 134	HB	1.84
			ASP 135	HB	2.07
				HB	2.54
			LEU 39	HB	1.84
			GLN 130	HB	1.99
			PHE 41	HB	1.84
				HB	1.91
11	Campesterol	−3.5982	No interaction		
12	Stigmasterol	−2.8915	ASP 135	HB	2.22
			GLY 44	HB	2.40
13	8 nonynoic acid	0.1127	OHE 41	HB	2.01
14	Beta sitosterol	0.4685	ASP 135	HB	1.97
			GLY 44	HB	2.48
15	9 Decynoic acid	1.1976	ASP 40	HB	1.88
			PHE 41	HB	1.86
16	Methyl non-8-ynoate	2.0473	PHE 41	HB	1.89
17	Methyl Dec-9-ynoate	2.8153	PHE 41	HB	1.95
18	Methyl 8-oxooctadec-9-ynoate	5.4298	PHE 41	HB	1.87
19	(9) Methyl (E)-11-methoxy-9-oxononadec-10-enoate	6.5759	PHE 41	HB	1.83
20	Methyl Sterculate	6.9274	PHE 41	HB	1.87
21	Methyl malvalate	8.0895	PHE 41	HB	1.87
22	**Orlistat**	**8.3009**	**ASP 40**	**HB**	**1.71**
			**GLU 134**	**HB**	**1.80**
			**GLY 44**	**HB**	**1.99**

Orlistat, as only standard drug used in market is used as standard reference for docking studies. Hence the docking result of orlistat in all tables is bold for ease of comparison.

**Table 7:** Summary of docking analysis with SCH1 protein (PDB ID 4XWX).

Sr. No.	Ligand	Dock score	Interacting residues	Bond type	Bond angle
1	Riboflavin	−13.553	ARG 74	Pi cation	5.25
				Pi Pi stacking	4.72
			ILE 150	HB	1.68
			ALA 153	HB	1.84
			SER 151	HB	1.95
				HB	2.19
2	Niacin	−11.0861	PHE 198	Pi-Pi Stacking	4.93
3	Ascorbic acid	−8.3129	ALA 153	HB	2.20
				HB	1.90
			SER 151	HB	1.58
				HB	1.88
4	Thiamine	−8.2065	ALA 153	HB	1.53
			PHE 198	Pi-Pi Stacking	4.09
			ILE 150	HB	1.73
5	Quercetin 3,3′ diglucoside	−6.2583	GLY 195	HB	1.94
			ALA 153	HB	1.58
			ILE 191	HB	2.10
				HB	2.49
			PHE 198	Pi – Pi Stacking	5.06
			SER 151	HB	2.24
			ILE 150	HB	1.61
				HB	1.75
6	Cyanidine 3, 5′ diglucoside	−4.9771	GLU 199	Salt Bridge	2.92
			PHE 198	Pi Pi Stacking	4.73
			ALA 153	HB	2.15
				HB	2.17
			SER 151	HB	1.84
			WATER	HB	2.43
			ILE 150	HB	1.81
				HB	1.77
7	Campesterol	−4.5453	ALA 153	HB	1.92
8	Beta sitosterol	−1.7076	ILE 191	HB	1.95
9	9 Decynoic acid	−1.6636	ARG 74	Salt Bridge	4.96
10	8 nonynoic acid	−1.5286	ARG 74	Salt Bridge	4.96
11	Stigmasterol	−1.0801	No interaction		
12	Quercetin 3,4′ diglucoside	0.3325	GLY 155	HB	2.43
			WATER	HB	2.17
			ALA 153	HB	1.88
				HB	2.07
				HB	2.33
			SER 151	HB	2.02
			PHE 198	Pi Pi Stacking	5.32
			GLY 195	HB	2.26
13	Beta rosasterol	0.3474	No interaction		
14	Methyl non-8-ynoate	0.477			
15	Methyl Dec-9-ynoate	1.0452			
16	Quercetin 3,7 diglucoside	2.2611	WATER	HB	1.14
				HB	0.61
				HB	2.50
			ILE 150	HB	1.76
				HB	1.66
			SER 151	HB	1.76
			ARG 74	Pi Cation	3.84
17	Methyl 8-oxooctadec-9-ynoate	3.4243	No Interaction		
18	Methyl malvalate	5.8575			
19	Methyl Sterculate	6.8808			
20	(9) Methyl (E)-11-methoxy-9-oxononadec-10-enoate	7.7443			
21	Cyanidin 3-sophoroside-5-glucoside	8.5222			
22	**Orlistat**	**10.3508**	**ALA 153**	**HB**	**1.86**

Orlistat, as only standard drug used in market is used as standard reference for docking studies. Hence the docking result of orlistat in all tables is bold for ease of comparison.

**Table 8:** Summary of docking analysis with ghrelin.

Sr. No.	Ligand	Dock score	Interacting residues	Bond type	Bond angle
1	Niacin	−11.1374	ALA 53	HB	2.36
			ASN 76	HB	1.85
2	Ascorbic acid	−7.2393	PRO 49	HB	2.15
				HB	1.86
			GLN 36	HB	1.77
			ALA 77	HB	2.18
3	Riboflavin	−7.0131	ALA 77	HB	2.40
			GLN 36	HB	1.68
				HB	1.59
			ASN 76	HB	1.62
4	Thiamine	−4.7344	GLN 36	HB	1.77
			ALA 77	HB	2.13
			HIE 32	Pi-Pi Stacking	5.33
5	8 nonynoic acid	1.9189	ASN 76	HB	1.84
6	9 Decynoic acid	2.7981	ALA 77	HB	2.13
7	Methyl non-8-ynoate	2.9037	No interaction		
8	Methyl Dec-9-ynoate	4.0035			
9	Campesterol	6.9115			
10	Methyl 8-oxooctadec-9-ynoate	8.7284	GLU 36	HB	2.20
			ASN 76	HB	1.92
11	Methyl malvalate	11.8293	ALA 77	HB	2.18
12	Methyl Sterculate	12.0917	No interaction		
13	Beta sitosterol	12.2015			
14	(9) Methyl (E)-11-methoxy-9-oxononadec-10-enoate	13.2915			
15	**Orlistat**	**15.8166**	**ASN 76**	**HB**	**1.65**
			**ALA 77**	**HB**	**2.20**

Orlistat, as only standard drug used in market is used as standard reference for docking studies. Hence the docking result of orlistat in all tables is bold for ease of comparison.

**Table 9:** Summary of docking analysis with MCH1.

Sr. No.	Ligand	Dock score	Interacting residues	Bond type	Bond angle
1	Quercetin 3,3′ diglucoside	−13.7266	ASP 91	HB	1.96
				HB	1.74
	
			GLY 80	HB	1.76
			GLY 18	HB	2.00
			SER 57	HB	1.73
2	Riboflavin	−12.9742	GLY 18	HB	2.19
				HB	2.10
			SER 87	HB	2.36
			SER 57	HB	1.55
3	Thiamine	−9.527	LEU 16	HB	1.82
				HB	1.90
			GLU 54	Salt Bridge	4.99
				HB	1.90
4	Quercetin 3,7 diglucoside	−8.7967	VAL 3	HB	2.02
			LEU 76	HB	1.63
			ACE 0	HB	2.10
			GLU 80	HB	2.24
			ASP 91	HB	2.35
5	Cyanidine 3, 5′ diglucoside	−8.3388	LEU 76	HB	1.68
			ACE 0	HB	1.63
				HB	1.51
			VAL 3	HB	2.31
			ASP 91	HB	1.55
				HB	1.58
			SER 87	HB	1.76
			GLY 18	HB	2.31
6	Ascorbic acid	−7.7733	SER 57	HB	2.19
				HB	1.80
			GLU 54	HB	1.76
				HB	1.65
7	Cyanidin 3-sophoroside-5-glucoside	−5.7144	LEU 76	HB	1.70
			ASP 91	HB	1.62
				HB	1.65
			GLY 18	HB	1.73
			ACE 0	HB	1.92
				HB	1.72
8	Quercetin 3,4′ diglucoside	−5.236	GLY 15	HB	2.09
			VAL 14	HB	2.35
			SER 57	HB	1.74
			SER 87	HB	2.20
			ASP 91	HB	1.56
				HB	1.42
9	Niacin	−5.127	VAL 3	HB	1.84
10	Campesterol	−0.843	GLU 54	HB	2.16
11	Stigmasterol	−0.787	GLU 54	HB	1.93
12	Beta sitosterol	1.849	GLU 54	HB	2.00
13	Beta rosasterol	2.848	No interaction		
14	8 nonynoic acid	3.749	VAL 3	HB	1.84
15	9 Decynoic acid	4.120	VAL 3	HB	1.84
16	Methyl Dec-9-ynoate	5.030	VAL 3	HB	1.89
17	Methyl non-8-ynoate	5.193	VAL 3	HB	1.89
18	(9) Methyl (E)-11-methoxy-9-oxononadec-10-enoate	8.373	TRP 61	HB	1.75
19	Methyl 8-oxooctadec-9-ynoate	8.384	SER 2	HB	2.03
20			VAL 3	HB	1.89
21	Methyl malvalate	11.003	VAL 3	HB	1.89
22	Methyl Sterculate	11.447	VAL 3	HB	1.89
23	**Orlistat**	**11.712**	**Glu 54**	**HB**	**2.09**

Orlistat, as only standard drug used in market is used as standard reference for docking studies. Hence the docking result of orlistat in all tables is bold for ease of comparison.

## 2 Experimental design, materials, and methods

### 2.1 Ligand preparation

Twenty two phytoconstituents present in *Hibiscus rosa-sinensis* were selected. Structures of all phytoconstituents were downloaded from PubChem database. Orlistat (PubChem CID 3034010) only available synthetic drug was used as reference standard.

### 2.2 Energy minimization

All structures were subjected to energy minimization using Avogadro software where universal force field (UFF) and first order steepest descent algorithm were used. This gave energetically stable conformations for the structures. Avogadro is free open source molecular builder software used for molecular modeling. It calculates the lowest energy conformation from the bond lengths and bond angles with smallest steric energy. Energy minimization helps in attaining structure conformation with lower delta G values which is considered close to biological system.

### 2.3 Retrieval of protein structure and preparation

Seven targets which play important role in maintaining energy balance of body and thus address obesity were selected. Protein structures of ligands were downloaded from the RCSB Protein Data Bank, database for 3D structures of large biological molecules, including proteins and nucleic acids. Downloaded protein structures were prepared X ray crystal structure of PDB ID 1LPB, 3LFM, 3TGZ, 1AX8, 4XWX for pancreatic lipase [2], FTO protein [3], cannabinoid receptor [4], hormones leptin [5] and protein SCH1 [6] respectively were selected. Data summarized in Table 1.

X- Ray crystal structure for Ghrelin [7] and MCH1 [8] receptor is not available in PDB databank so model protein structure was created using I-TASSER server online. FASTA sequence was taken from Uniprot ID of protein and submitted for model preparation. Table 2 summarizes FASTA sequence of Ghrelin and MCH1. Model was evaluated for C-score, TM score and RMSD. Model with C-score between −5 and 2, TM score greater than 0.5 were selected. Finalized model were validated on PROSA, Saves v5.0, Ramachandran plot and ProQ and then were used as receptors.

### 2.4 Molecular docking studies

Molecular docking techniques dock small molecules into the protein binding site. In order to understand how these ligands bind to the enzyme, docking analysis were performed using FlexX software. The receptor ligand interactions were done using Maestro software. Interacting amino acid residue, bond type and bond distance were noted.

Data summarized in Table 3, Table 4, Table 5, Table 6, Table 7, Table 8, Table 9 and Fig. 1, Fig. 2, Fig. 3, Fig. 4, Fig. 5, Fig. 6, Fig. 7, Fig. 8, Fig. 9, Fig. 10, Fig. 11, Fig. 12, Fig. 13, Fig. 14.
