# Supplementary material for: Computational data of phytoconstituents from Hibiscus rosa-sinensis on various anti-obesity targets
Source: Data Brief. 2019 May 16;24:103994. doi: 10.1016/j.dib.2019.103994 (PMC6538924; doi:10.1016/j.dib.2019.103994)
Supplement: Multimedia component 1 [file mmc1.docx]

**Conflict of Interest:** None to declare
